# DNA Microarray for Rapid Detection and Identification of Food and Water Borne Bacteria: From Dry to Wet Lab

**DOI:** 10.2174/1874285801711010330

**Published:** 2017-11-30

**Authors:** Reza Ranjbar, Payam Behzadi, Ali Najafi, Raheleh Roudi

**Affiliations:** 1 Molecular Biology Research Center, Baqiyatallah University of Medical Sciences, Tehran, Iran; 2 Department of Microbiology, College of Basic Sciences, Shahr-e-Qods Branch, Islamic Azad University, Tehran, Iran; 3 Oncopathology Research Center, Iran University of Medical Sciences, Tehran, Iran

**Keywords:** DNA Microarrays, DNA Microchips, Bioinformatics, DNA Probes, Bacteria

## Abstract

**Background:**

A rapid, accurate, flexible and reliable diagnostic method may significantly decrease the costs of diagnosis and treatment. Designing an appropriate microarray chip reduces noises and probable biases in the final result.

**Objective:**

The aim of this study was to design and construct a DNA Microarray Chip for a rapid detection and identification of 10 important bacterial agents.

**Method:**

In the present survey, 10 unique genomic regions relating to 10 pathogenic bacterial agents including *Escherichia coli (E.coli), Shigella boydii, Sh.dysenteriae, Sh.flexneri, Sh.sonnei, Salmonella typhi, S.typhimurium, Brucella sp., Legionella pneumophila,* and *Vibrio cholera* were selected for designing specific long oligo microarray probes. For this reason, the in-silico operations including utilization of the NCBI RefSeq database, Servers of PanSeq and Gview, AlleleID 7.7 and Oligo Analyzer 3.1 was done. On the other hand, the **in-vitro** part of the study comprised stages of robotic microarray chip probe spotting, bacterial DNAs extraction and DNA labeling, hybridization and microarray chip scanning. In wet lab section, different tools and apparatus such as Nexterion® Slide E, Qarray^mini^ spotter, NimbleGen kit, TrayMix^TM^ S4, and Innoscan 710 were used.

**Results:**

A DNA microarray chip including 10 long oligo microarray probes was designed and constructed for detection and identification of 10 pathogenic bacteria.

**Conclusion:**

The DNA microarray chip was capable to identify all 10 bacterial agents tested simultaneously. The presence of a professional bioinformatician as a probe designer is needed to design appropriate multifunctional microarray probes to increase the accuracy of the outcomes.

## 1 INTRODUCTION

Food and water borne pathogenic bacteria including *E. coli, Shigella, Salmonella, Vibrio cholera, Brucella and Legionella *are considered as the most important causes of life-threatening diseases throughout the world. The infections caused by these bacterial pathogens are endemic now in many countries including Iran [1-7].

The conventional methods for detection of bacterial infections may take several days to be completed. In recent years, DNA-based approaches have become potentially powerful in microbiological diagnostics and molecular epidemiology of infections because of their higher rapidity, reproducibility, accuracy, and affordability [8, 9].

DNA microarray technology is a proper and high-throughput diagnostic tool for detection and identification pathogenic microorganisms. It is flexible, sensitive, specific and very sharp technique. The process of this technology is divided into two sections of dry laboratory (in-silico) (Bioinformatic practices) and wet laboratory (*in-vitro*). The dry laboratory of microarray technique includes the processes of probe designing and finalizing data analysis which are known as in-silico study. Other stages of DNA microarray technology such as probe spotting, DNA labeling, and hybridization are recognized as wet laboratory (*in-vitro*) practices [10-18].

Traditional microbiological, immunological and biochemical assays are generally time consuming and they normally give some false +ve or –ve results. Moreover, the application of advanced molecular techniques such as Polymerase Chain Reaction (PCR) is useful when the number of samples is low and limited. In the presence of a huge number of clinical/environmental samples, DNA microarray technology is a suitable option as a rapid and reliable diagnosis [16, 19-26]. To construct a DNA microarray chip, the designed single stranded probes are immobilized on the solid surface of a chip by an automated and robotic spotting machine (spotter/printer). The immobilization of DNA microarray long oligo probes is done when the probes are modified by different types of active chemical groups such as NH_2_, Succinyl, Disulfide, and Hydrazide. Originally, the microarray chip is made of glass; but, the surface of a chip may be treated with different types of chemical materials including Epoxy silane, Isothiocyanate, Aminophenyl, Mercaptosilan, Aldehyde or Epoxide to improve the stability of the covalent bonds between a single stranded DNA probe and the surface of a chip. The modified probes and the related treated chips guarantee the occurrence of a stable, powerful and optimized bond. Each chemical group in a modified probe needs its specific chemical substance on the treated chip slides. For example, the NH_2_ group in a modified probe binds best with the Epoxy silane covered chip and they create powerful covalent bonds [10, 16, 17, 27-30].

In parallel with the probe spotting, the bacterial cells isolated from samples must be handled in the process of DNA extraction. If the bacterial cells are few in clinical samples, there are two different ways to prepare an appropriate amount of bacterial DNA molecules: 1. Performance of PCR, 2. Utilization of culture medium. Regarding E.coli, both ways are possible but not for fastidious or unculturable bacteria. Then, the extracted DNAs must be melted into single strands and labeled with fluorescent dyes such as Cy3, Cy5, Tamro, and Texas Red. In the field of microbial diagnosis, the use of one color (one-channel microarray) is acceptable while, regarding gene expression profiling the use of two fluorescent dyes is needed (two-channel microarray). In the third step, labeled bacterial DNAs (target sequences) and immobilized probes go through the hybridization process. Finally, the hybridized DNA strands (probes and labeled targets) result in fluorescent spots which are detectable by the microarray scanner. In this survey, the authors have designed and constructed a DNA microarray chip for detection and identification of 10 bacterial pathogens.

## 2 MATERIALS AND METHODS

### 2.1 Bacterial Samples and Culture Medium (Wet Lab)

In the present investigation, clinical bacterial samples including *E. coli, Sh. boydii, Sh. dysenteriae, Sh. flexneri, Sh. sonnei, Salmonella typhi, S. typhimurium, Brucella sp., L. pneumophila,* and *V. cholera* were aerobically inoculated in Luria-Bertani (LB) agar and incubated at 37°C for 48 hours. After a suitable growth of bacterial colonies, the total DNA molecules were extracted. The accuracy of the bacterial samples was confirmed by biochemical tests [31-34].

### 2.2 DNA Extraction Procedure (Wet Lab)

The process of total DNA extraction was performed in accordance with the modified boiling water protocol suggested by Peng *et al.* [35]. So, the bacterial colonies were scratched from LB agar and put into a microtube containing 100 μl double distilled water (ddw). The microtube was shaken for dissolving bacterial cells and then was put into the boiling water vertically for 10 minutes. The tube was floating in the boiling water via the cork. In the next step, the microtube containing bacterial cells and ddw was centrifuged for 10 minutes at 13000 rpm. Finally, the supernatant containing DNAs was put into a new sterile microtube for the following steps and the pellet was omitted. The DNA purity was checked via nanodrop spectrophotometer. The appropriate OD range is reported from 1.7 to 2.0. These processes were done for each bacterial sample, separately [36].

### 2.3 Microarray Long Oligo Probe Designing (Dry Lab)

Each of the long oligo microarray probe was designed and checked by the authors. In brief, the complete genome data for each bacterial sample was downloaded from NCBI ftp and RefSeq complete genome. Then, the related .gbk and .fna files pertaining to each bacterial sample and its close phylogenetic microorganisms were compared by GView server. In this study the PanSeq Server, BLASTn tool, AelleID 7.7 software, and OligoAnalyzer tool were also employed for designing and analyzing the long oligo microarray probe [13, 16-18, 37].

In accordance with the aforementioned methodology, we just got a single one microarray long oligo microarray probe with the Best Quality (Table **1**).

The designed probes were ordered by Bioneer Company (South Korea) for synthesis. Besides, the probes were modified by its 5' end with a six-carbon-spacer and a NH_2_ group [27, 38].

### 2.4 DNA (Target Sequence) Labeling (Wet Lab)

The extracted total DNAs were amplified and labeled in accordance with NimbleGen (Roche) protocol [39].A single color of Cy3 as one-channeled DNA microarray was used in the present study. The concentration of each bacterial DNA (including *E. coli, Sh. boydii, Sh. dysenteriae, Sh. flexneri, Sh. sonnei, V. cholerae, S. typhi, S. typhimurium, Brucella sp.,* and* L. pneumophila,*) was adjusted. So, the related amounts were taken from each bacterial DNA respectively, put into an RNase and DNase free microtube and mixed with each other. 40 μl Cy3 (the cyanine dyes) was added to the mixed bacterial genomes. Finally, the PCR grade water was added to the mixture of Cy3 and bacterial genomes. The total volume of the mixture was reached up to 80 μl. This process was performed in a dark room for avoiding Cy3 destruction. The final mixture was completely mixed through up-and-down performance and incubated in PCR thermocycler at 98°C for 10 minutes. Right after, the incubated mixture was put into the ice for 2 minutes.

Then, a mixture of 8 μl PCR grade water, 10 μl dNTP and 2 μl Klenow fragment (with a total volume of 20 μl) was added to the fluorescent bacterial genomic DNA. The final mixture with the volume of 100 μl was completely dissolved by up-and-down technique. Next, it was incubated in PCR thermocycler at 37°C for 2h and in follow, 21.5 μl stopsolution and 110 μl isopropanol were respectively added to the incubated mixture. The latter was incubated at lab temperature (25°C) for 10 minutes. After incubation, the mixture was centrifuged at 12000 rpm for 10 minutes. The supernatant was discharged and 500 μl from cold ethanol 80% was added to the pellet. The pellet was suspended within the ethanol and then centrifuged at 12000 rpm for 2 minutes. Again, the supernatant was discharged and the microtube was put opened. The opened and dried microtube was centrifuged by the DNA vacuum concentrator at 3000 rpm for 5 minutes. Finally, 25 μl PCR grade water was added to the bacterial DNA mixture pellet and its OD was measured by nanodrop spectrophotometer as 543 nm.

### 2.5 Probe Spotting on Microarray Chip (Wet Lab)

According to the SCHOTT protocol, 17X30 μl containing 15 μl 50 pmol of designed long oligo microarray probes and 15 μl spotting buffer were added to 14 wells (A1-A2, B1-B2, C1-C2, D1-D2, E1-E2, F1-F2, and G1-G2) of a 384-well microplate. The 3 other wells including F2, G1-G2 were filled by spotting buffer as the blank controls in a layout of 2X4. The microplate and epoxy silane covered chip (Nexterion^®^ Slide E, SCHOTT, Germany) were placed into the Qarray^mini^ spotter (Genetix, UK)). The spotter was programmed by Qarray software and the DNA probes were spotted onto Nexterion^®^ Slide E in quadruplicate at 25 °C and 45% humidity. So, the probe was printed in a layout of 4X2 on the chip for quadruplicate [40].

A1-A2, B1-B2, C1-C2, D1-D2, E1-E2, F1-F2, and G1-G2 wells were filled by the designed long oligo microarray probes. The F2, G1 and G2 wells were filled by spotting buffer as blank control samples. The spotted chip was incubated at lab temperature in 30 minutes and then was incubated in humidity chamber with 45% humidity for an overnight. Then, the spotted chip was washed to remove the unchained printed probes. The unfastened probes might be the reason for interference and noise in hybridization process. The chip was rinsed at lab temperature with three different buffer substances including 0.1% Triton^®^ X-100 (once for 5 minutes), 1 mM HCl solution (twice for 2 minutes), 100 mM KCl solution (once for 10 minutes), respectively. The washed printed chip was then incubated in blocking solution at 50°C for 15 minutes to fasten bound probes. The treated chip was then rinsed by deionized water (DIW) at lab temperature for 1 minute. Finally, the spotted Nexterion^®^ Slide E was dried via centrifugation at 1300 rpm for 5 minutes [40-42].

### 2.6 Hybridization and Analysis Processes (Wet Lab)

The hybridization process was performed by TrayMix^TM^ S4 hybridization station (BioTray). 2 μl of the labeled bacterial DNA mixture was mixed with 8 μl PCR grade water within a microtube. The 10 μl-volume-mixture was then incubated in PCR thermocycler at 95°C for 3 minutes. The incubated mixture was added into TrayMix^TM^ S4 and processed according to the programmed software. The prehybridization process was done at 42°C for 5 minutes. Right after, the hybridization process was performed for 3.5 hours by Nexterion^®^ Hyb at 42°C. By the end of hybridization process, the post hybridization rinsing was achieved by TrayMix^TM^ S4 at 25 °C. The hybridized chip was rinsed by solution I (2XSSC + SDS 0.2%) (once for 10 minutes), solution II (2XSSC) (once for 10 minutes), and then solution III (0.2XSSC) (once for 10 minutes), respectively. At the end, the spotted Nexterion® Slide E was dried via centrifugation at 1300 rpm for 5 minutes [40, 41]. The dried chip was scanned at the resolution of 10μm by Innoscan 710 and Mapix software (Innopsys, Midi-Pyrénées area of France). All the procedures were performed thrice per genome.

## 3 RESULTS

In this study the best designed microarray probes were successfully worked (Table **1**).

**Fig. (1):**
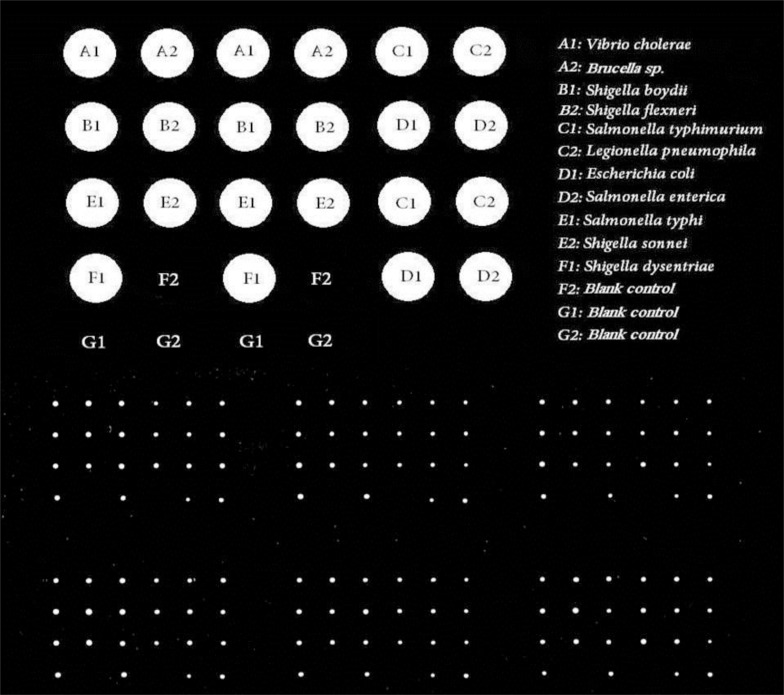
Resolution of 10μm by Innoscan 710. The labeled bacterial DNAs matched with their specific designed probe (successful hybridization); while, the control spots (blank spotting buffer) shows no successful match with the spotted probe. There are 6 arrays of 24-spotted array which is illustrated in Fig. (**1**).

It is clear that, the probes which were recognized as the Best Options by AlleleID software were selected. The scanning results confirmed the qualified specificity of the probe. As Fig. (**1**) indicates, the Cy3 labeled genomic DNAs belonging to bacterial samples had successful hybridization. This result confirms the qualified specificity of the designed microarray long oligo probe. No hybridization has happened in blank controls (Fig. **1**).

## 4 DISCUSSION

A rapid and reliable diagnostic technique is the main goal of the clinical laboratories' experts. Simultaneously, sensitivity, specificity and reproducibility are other preferred options in clinical diagnostics. Among several molecular biology tools, DNA microarray technology is a proper and appropriate technique which enables us to detect and identify a wide range of pathogenic and non-pathogenic microbial agents at once. As the pan-genomic technology of microarray is able to reveal a huge number of genomic content dissimilarities within determined microbial strains, it seems to be an invaluable tool for detection and identification of close microbial agents [43-48]. There are different types of nucleic acid based techniques like conventional PCR, multiplex PCR and etc.; but when there are a huge number of specimens so; PCR techniques will be expensive and time consuming. The sensitivity, specificity and accuracy of the DNA microarray techniques are too sharp. Construction and designing a local microarray chip makes it flexible and effective. Because when a microarray chip is designed in a local lab, it can be useful to be upgraded for genotyping. This idea makes the microarray technology cheap, reliable and accessible. In other words, DNA microarray technology is known as a lab on chip technique which is applicable for several thousand specimens at once [10, 13, 14, 16, 18, 23, 37, 49].

The previous studies report that the sensitivity of microarray is determined by DNA labeling kit. It ranges between 1.0 ng and 10 μg DNA. In our study, the NimbleGen (Roche) kit was used for bacterial DNA labeling; which its sensitivity was 1 μg DNA [39, 43, 50].

By the use of DNA microarray we were able to detect and identify the bacterial samples within 3 days which was significantly shorter than several clinical techniques [43].

The in-silico part of microarray technique which is in association with microarray probe designing has a considerable importance on the quality of the microarray outcomes. In other words, the ability of probe designer in association with microbial phylogenetic relationships and Bioinformatics determines the accuracy and reliability of microarray tool results [10, 14, 16, 18, 37, 38, 41, 46].

The use of microarray long oligo probes increases the specificity of microarray probes which may lead to have a high quality outcome. For this reason we designed a long oligo probe with a length of 58 nucleotides [46, 51].

The pan-genomic diagnostic tool of microarray is based on genes and other genomic elements; thus selection of different unique genes as suitable target sequences increases the sharpness of the work. So, in the present study, the unique genes were significantly selected for each bacterial agent [32, 38, 44].

## CONCLUSION

Our chip was capable of identifying all 10 bacterial agents tested simultaneously.The knowledge of Bioinformatics affects directly on the quality of microarray technology. As the result, there is a very interesting possibility for detecting and identifying bacterial agents and even antibiotic resistant strains. Therefore, the art of probe designing makes DNA microarray tool flexible and powerful diagnostic method with high accuracy, sensitivity and specificity within the shortest time.

## Figures and Tables

**Table 1:** A microarray probe designed by AlleleID 7.7 for unique regions.

**Related Gene**	**Length**	**Designed Long Oligo Microarray Probes** o ** (5'→3')**	**Microorganisms**
NC_010740.1; 564401-564458/ twin-arginine translocation pathway signal protein	58	**GTCCTCATGCGAGCGTTTCATCGTTGCGGTCAGGTCTTCTAAAGAATAGTTCAACTGG**	***Brucella* sp.**
NC_010468.1; 4619941-4619998/ TetR family transcriptional regulator	58	**AAATAGCCGCCAGTTGTGTATTATTCATATTTGAACTTATTTCGCCTGAAGATAACGC**	***E. coli***
NC_006369.1; 2041551-2041612/ putative Apyrase	62	**AAATGCGGAGATTAGCAAACATAACCAGGTAGAACTCAATATCTATGGACAGAATATCAATC**	***L. pneumophila***
1877374-1877430/ exported phage protein	57	**TTTGAGTAACTAATTGAAACCTTGGTATATCCAGACTGCTGCCCTTTCTTTCTTAAA**	***S. enterica***
NC_003198.1; 4534547-4534606/ hypothetical protein	60	**CGACCATTGAACCGACAATCTTGCTTATTCCATTACGACAATCACATTCATAGGATTCTT**	***S. typhi (37)***
NC_003197.1; 2866027-2866084/ hypothetical protein	58	**CGGACTCTAACTCTCTAGGGCTCTTATATATTCCTGTGGCAACATATTTAGCGGTAAA**	***S. typhimurium***
NC_007613.1; 3805070-3805132/233 bp at 5' side: single-stranded DNA-binding protein80 bp at 3' side: hypothetical protein	63	**CCCTGTTGTCTCAAAATGGCTAAATGTTGTCTCGGTGTTGTCTCAGAAATTAAAATAAAATCC**	***Sh. boydii***
NC_007606.1; 3769778-3769835/ hypothetical protein	58	**AATATGATAATACCGTTTCCGTGGGTAGCAGCACTCTACAGCGTAAAGTCGTCAATAT**	***Sh. dysentriae***
NC_004741.1; 3520449-3520506/chaperone,Capsule protein fraction 1,pili assembly chaperone protein SafB,Gram-negative pili assembly chaperone, N-terminal domain	58	**GCCAGGATATTAAATGGTCTGTGATTACTGATGAAGGTGGTGAGAGTCGTTTGTTTAT**	***Sh. flexneri***
NC_007384.1; 1664092-1664150 / putative methylase	59	**GCTTGGATGAGTTTGGTATTGCTCGAATCTGTACTAATGCCACTATGTGTAAAGGTAAA**	***Sh. sonnei***
NC_012578.1; 15648-15704/ 10 bp at 5' side: _nteric oxidoreductase, YhdH/YhfP family protein182 bp at 3' side: threonine ammonia-lyase, biosynthetic	57	**CCCTCTGTTATGCCTTTTCTACCTGAATGGACAAGTAGTCGGGTATGGATGGTAAAG**	***V. cholerae***
